# The Full Region of N-Terminal in Polymerase of IBDV Plays an Important Role in Viral Replication and Pathogenicity: Either Partial Region or Single Amino Acid V4I Substitution Does Not Completely Lead to the Virus Attenuation to Three-Yellow Chickens

**DOI:** 10.3390/v13010107

**Published:** 2021-01-14

**Authors:** Weiwei Wang, Yu Huang, Zhonghua Ji, Guo Chen, Yan Zhang, Yuanzheng Qiao, Mengya Shi, Min Li, Teng Huang, Tianchao Wei, Meilan Mo, Xiumiao He, Ping Wei

**Affiliations:** 1 Institute for Poultry Science and Health, Guangxi University, Nanning 530005, China; wangweiweihn@163.com (W.W.); 15770938819@163.com (Y.H.); jizhonghua9@126.com (Z.J.); gchen8@126.com (G.C.); yanzhang8812@163.com (Y.Z.); qiaoyuanzheng1996@126.com (Y.Q.); mengyashi12@126.com (M.S.); liminmingd@163.com (M.L.); tenhhwang@163.com (T.H.); tcwei88@126.com (T.W.); momeilan@163.com (M.M.); 2 School of Marine Sciences and Biotechnology, Guangxi University for Nationalities, Nanning 530006, China; 3 Guangxi Key Laboratory Cultivation Base for Polysaccharide Materials and Modifications, Guangxi University for Nationalities, Nanning 530006, China

**Keywords:** Infectious Bursal Disease Virus, N-terminal domain of VP1, reverse genetics technique, recombinant virus, viral replication and pathogenicity

## Abstract

Infectious Bursal Disease Virus (IBDV) has haunted the poultry industry with severe, prolonged immunosuppression of chickens when infected at an early age and can easily lead to other secondary infections. Understanding the pathogenic mechanisms could lead to effective prevention and control of Infectious Bursal Disease (IBD). Evidence suggests that the N-terminal domain of polymerase in segment B plays an important role, but it is not clear which part or residual is crucial for the pathogenicity. Using a reverse genetics technique, a molecular clone (rNN1172) of the parental vvIBDV strain NN1172 was generated, and its pathogenicity was found to be the same as the parental virus. Then, three recombinant chimeric viruses were rescued based on the rNN1172 and substituted with the counterparts in the N-terminal domain of the attenuated vaccine strain B87: the rNN1172-B87VP1a (substituting the full region of the 1–167 aa residuals), the rNN1172-B87VP1a∆4 (substituting the region of the 5–167 aa residuals), and the rNN1172-VP1∆4 (one single aa residual substitution V4I), to better explore the role of the N-terminal domain of polymerase on the viral pathogenicity. Interestingly, all these substitutions played different roles in the viral pathogenicity: the mortality of the rNN1172-B87VP1a-challenged chickens was significantly reduced from 30% to 0%. No obvious lesion was found in the histopathological examination, and the lowest viral genome copy number was also detected in the bursa when compared to the parental and two other recombinant viruses. The mortalities caused by rNN1172-B87VP1a∆4 and rNN1172-B87VP1∆4, respectively, were all reduced to 10% and had a delayed onset of death. Our results also revealed that the pathogenicity of the IBDV was consistent with the viral replication efficiency in vivo (bursae). This study demonstrated that the full region of the N-terminal of polymerase plays an important role in viral replication and pathogenicity, but the substitutions of its partial region or a single residual do not completely lead to the virus attenuation to Three-Yellow chickens, although that significantly reduces its pathogenicity.

## 1. Introduction

Infectious Bursal Disease Virus (IBDV), a member of the genus *Avibirnavirus* of the family Birnaviridae, is a major pathogen causing immunosuppressive disease in chickens and is economically significant to the poultry industry worldwide [1]. Two different serotypes of IBDV have been described: the pathogenic serotype 1 can cause long-term immunosuppression and death, while serotype 2 isolates are not pathogenic to chickens [2,3]. In the 1970s, the classic IBDV (cIBDV) was first described [4]. Subsequently, increasing numbers of “antigenic variant IBDV” (avIBDV) and “very virulent IBDV” (vvIBDV) have been isolated from vaccinated and unvaccinated chicken flocks [5,6,7,8].

The IBDV genome contains two segments (A and B) [9,10]. Segment A encodes nonstructural protein VP5, capsid protein VP2, scaffold protein VP3, and protease VP4 [11,12,13,14,15]. Segment B encodes VP1, an RNA-dependent RNA polymerase (RdRp) responsible for the replication of the viral genome and synthesis of mRNAs [16]. In recent years, more and more mosaic/novel variant IBDVs have been reported, which may have evolved by reassortment events of the two genome segments [17,18,19], by homologous recombination within segments [20,21,22], and by gene mutations in the hypervariable region of VP2 (*v*VP2) [23,24]. Especially, since 2017, more and more novel variant strains with great immunosuppressive effects in chickens emerged in most parts of China [23]. These were isolated from vaccinated chicken flocks in which the classical vaccines had been proven ineffective at controlling novel variant strains [25,26]. The mechanisms of these changes are not fully understood. Some studies note, for example, the VP5 protein promotes virion release from infected cells [13,27] and does not cause cell death [28] and bursal lesions [29]. The VP4 protein contributes to the cleavage of intermediate precursor VP4-VP3 polyprotein [30]. VP3, as a scaffolding protein, plays a critical role in capsid assembly [31]. The VP2 protein plays important roles in virulence and as a host-protective immunogen, and changes within the neutralizing epitopes are responsible for antigenic variation [32,33,34]. It was also confirmed that VP2 is not the sole determinant of the very virulent phenotype [35]. All these studies failed to fully elucidate the pathogenesis of the virus.

Our group and others have confirmed that the genomes of the wild-type IBDVs in chickens have been constantly changing and that multiple types of reassortant strains are prevalent in the field [7,36,37,38,39,40,41]. The VP1 protein of IBDV has been confirmed to be involved in the efficiency of viral replication and modulates the virulence in vivo [42,43]. Other studies indicate that VP1 of segment B may play a role in viral pathogenesis [44,45,46]. The VP1 of IBDV is an 878 amino acid (aa) protein that could be divided into the N-terminal domain (designated as a, 1–167 aa), the central polymerase domain (designated as b, 168–658 aa), and C-terminal domain (designated as c, 659–878 aa) [47,48], and it is not fully clear which regions or sites are important for its pathogenicity. Nouen et al. found that several regions of segment B of IBDV are required for viral pathogenicity [49]. As suggested by Gao et al., different domains of VP1 have different roles in virus replication and virulence, and the N-terminal may play a more important role [50]. In addition, Yu et al. have confirmed that the V4I substitution in the N-terminal of VP1 can affect viral replication and pathogenicity [51]. Although some progress has been made in understanding the molecular biology of IBDV, the molecular basis for the viral pathogenicity has not yet been fully defined. In order to clarify which part or single residual in the N-terminal domain of VP1 affects the pathogenicity of the virus, we used the reverse genetics technique to substitute the full region, the partial region, and the single aa residual of the VP1 N-terminal domain and evaluated their pathogenicity in commercial Three-Yellow chickens.

## 2. Materials and Methods

### 2.1. Embryos, Cells, and Viruses

Chicken embryo fibroblasts (CEFs) were prepared from the 9-day-old embryonated specific-pathogen-free (SPF) chicken eggs (Beijing Merial Vital Laboratory Animal Technology Co., Ltd., Beijing, China) according to the conventional method as noted in our previous study [52]. vvIBDV strain NN1172 was isolated from the field [53] and kept in our laboratory. The commercial attenuated IBDV vaccine strain B87 was purchased from the local vaccine market. Viruses were propagated in the 9-day-old SPF embryonated chicken eggs and titrated, expressed as median embryo lethal doses (ELD_50_), according to the previously described method [54].

### 2.2. Construction of the Full-Length cDNA Clones of NN1172′s Segments A and B

The RNA polymerase II reverse genetic system previously established by Qi et al. [55] was used to rescue the IBDV in our study. Briefly, the full-length A and B segments of the NN1172 strain were amplified with the primers (Supplementary Material Table S1) according to the previous report [56]. The hammerhead ribozyme (HamRz) and hepatitis delta ribozyme (HdvRz) cDNA sequences were fused with segment A or segment B of NN1172 with the primers (Supplementary Material Table S2) in three steps by multiple PCR as described previously [57], resulting in the clones pMD18T-mNN1172AIII and pMD18T-mNN1172BIII, respectively.

Each segment, fused with HamRz and HdvRz, was then cloned into the downstream CMV enhancer and T7 promoter of pVAX1 vector by homologous recombination according to the manufacturer’s instructions in the ClonExpress^®^ MuitiS One Step Cloning Kit (Vazyme, Nanjing, China) with the primers shown in Table 1. First, the pVAX1 plasmid was linearized by using *EcoR* I single enzyme digestion. Second, using pMD18T-mNN1172AIII and pMD18T-mNN1172BIII as a template, both ends of segments A and B were fused with the homologous sequences using primers A3U/B3U and A3L/B3L, respectively, to obtain NN1172A3 and NN1172B3. Finally, the homologous recombination of NN1172A3 and NN1172B3 with the linearized pVAX1 vector was carried out respectively (5× CE MultiS Buffer 2 μL, linearized pVAX1 vector 60 ng, NN1172A3/NN1172B3 69/62 ng, Exnase MultiS 1 μL, ddH_2_O up to 10 μL; 37 °C water bath for 30 min). The resultant plasmids containing the segment A or B sequence were designated pVAX1NN1172A and pVAX1-mNN1172B, respectively.

**Figure 1:**
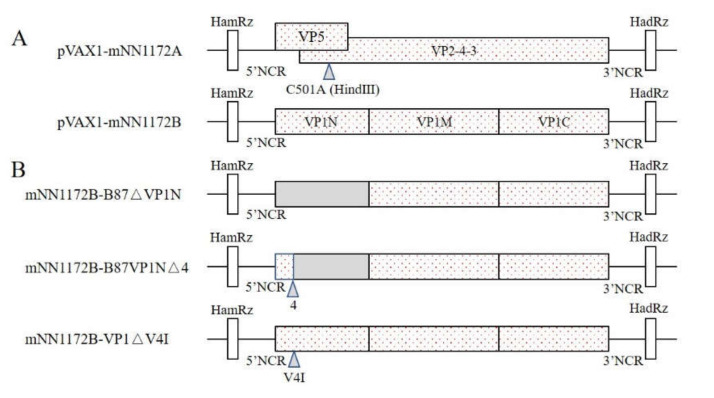
Schematic representation of the unique restriction enzyme site (*Hind* III) was introduced into the Infectious Bursal Disease Virus genome (**A**) and construction of mosaic B segments derived from NN1172 (open boxes) and B87 (shaded boxes) (**B**).

A unique restriction enzyme site (*Hind* III) was introduced into the plasmid pVAX1-mNN1172A by a silent mutation (C501A) of the polyprotein as genetic tags for the rescued virus (Figure 1A), according the manufacturer’s instructions in the Mut Express^®^II Fast Mutagenesis Kit V2 (Vazyme, Nanjing, China). First, the pVAX1-mNN1172A plasmid was amplified with primers ADTB-F/ADTB-R and Phanta Max super-Fidelity DNA polymerase to obtain the linearized vector with mutation introduction (2× Max Buffer 12.5 μL, dNTP Mix 0.5 μL, pVAX1-mNN1172A 1.5 μL, each of ADTB-F/ADTB-R 1μL, Phanta Max Super-Fidelity DNA Polymerase 0.5 μL, ddH_2_O 5 μL; 30 cycles with denaturation at 95 °C for 15 s, annealing for 15 s at 60 °C, and extension at 72 °C for 6 min; and then a final elongation step at 72 °C for 5 min). Because the 5′ ends of primers ADTB-F and ADTB-R contain a complete reverse complementation sequence, the linearized plasmid with the introduced mutation could be used to finish plasmid cyclization by homologous recombination with Exnase II (5× CE Buffer 2 μL, *Dpn* I digestion products 0.5 μL, Exnase II 1 μL, ddH_2_O 6.5 μL; 37 °C water bath for 30 min), to obtain pVAX1-mNN1172A.

### 2.3. Construction of Mosaic B Segments

The new mosaic B segments were constructed by introducing different regions of the N-terminal domain of B87 strain under the background of pVAX1-mNN1172B: (i) the substitution of the whole nucleotides encoding the N-terminal domain (designated as B87VP1a, 1–167 aa residuals in the N-terminal domain of the viral RNA polymerase of NN1172 with the corresponding part of the attenuated vaccine strain B87), (ii) substituting the region of the 5–167 aa residuals in the N-terminal domain of the viral RNA polymerase of NN1172 with the counterpart’s residuals of B87 (designated as B87VP1a∆4), and (iii) one single aa residual substitution V4I (by the nucleotide substitution G10A) in the N-terminal domain of the viral RNA polymerase of NN1172 (designated as VP1∆4) (Figure 1B).

In order to construct a full-length clone of segment B of NN1172 with the VP1a of B87 strain, the vector was first linearized from pVAX1-mNN1172B by reverse PCR using the primers ZTB87NF/ZTB87NR (30 cycles with denaturation at 98 °C for 10 s, annealing for 15 s at 55 °C, and extension at 72 °C for 3 min). Second, the B87VP1a was amplified with primers B87NF/B87NR (30 cycles with denaturation at 98 °C for 10 s; annealing for 15 s at 55 °C and extension at 60 °C for 30 s) and replaced by its counterpart similarly by homologous recombination according to the manufacturer’s instructions in the ClonExpress^®^ MuitiS One Step Cloning Kit (Vazyme, Nanjing, China) (5× CE MultiS Buffer 2 μL, linearized vector 109 ng, B87VP1a 20 ng, Exnase MultiS 1 μL, ddH_2_O up to 10 μL; 37 °C water bath for 30 min), to obtain mNN1172-B87VP1a. To construct a clone of segment B of NN1172 with the VP1a∆4 of B87 strain, the vector was first linearized by reverse PCR using the primers ZTB87NF/ZTB87NBFR, and the VP1a∆4 fragment was amplified with primers B87NBFF/B87NR and replaced by its counterpart similarly derived from the B87 strain by the same methods as above, to obtain mNN1172-B87VP1a∆4. To obtain mNN1172-VP1∆4 plasmids, the V4I substitution of the polymerase was introduced into the pVAX1-mNN1172B plasmid by site-directed mutagenesis with primers WD4TBF/WD4TBR according to the manufacturer’s instructions in the Mut Express^®^II Fast Mutagenesis Kit V2 (Vazyme, Nanjing, China) as described above.

### 2.4. Transfection on the Primary CEFs to Rescue the Viruses

CEFs were grown in six-well cell culture plates to 85–95% confluence and were co-transfected with various combinations (shown in Table 2) by using Invitrogen Lipofectamine^TM^ 2000 reagent (Invitrogen, Carlsbad, CA, USA) according to the manufacturer’s protocol. Briefly, after removing the DMEM supernatants from CEFs, each of the plasmids containing mixtures was added gently to CEFs. After 6–8 h, the transfection mixture was replaced with 2 mL of fresh DMEM containing 10% FBS and then continually incubated at 37 °C/5% CO_2_ for 72 h, respectively. After 72 h of incubation, in order to evaluate the existence of infectious virus in transfected CEFs, the CEFs were freeze-thawed three times, and then the supernatants of each transfection were collected and inoculated into 9-day-old SPF chicken embryo eggs (0.4 mL/embryo) via the chorioallantoic membrane (CAM) route, respectively. Embryos were incubated at 37 °C and monitored twice daily for 3–5 days. Then the allantoic fluids, CAM, and the embryo tissues were collected, homogenized, and the harvested suspensions were freeze-thawed three times before being used for the passage in the embryos for another two continual passages as described above. The final harvested homogenates were stored at −80 °C until used. The successful rescued viruses were named *r*NN1172, *r*NN1172-B87VP1a, *r*NN1172-B87VP1a∆4, and *r*NN1172-VP1∆4, respectively.

### 2.5. Identification of the Rescued Viruses

The RNA extraction and reverse transcriptase reactions of the homogenate samples of the inoculated embryos of the 3rd passage were performed according to our previously described methods [36]. The full-length genomes (segments A and B) of the rescued viruses were amplified and sequenced as described previously [56]. The successfully rescued virus should contain the genetic tag of restriction enzyme *Hind* III (C501A) site, which was only present in the A-segment cDNA plasmid (pVAX1-mNN1172A). Enzymatic digestion of the purified PCR products with *Hind* III (segment A) can be used to identify the rescued virus. In addition, the HiFi script reverse transcriptase (Cwbio, China, Beijing) was left out from one sample to determine that the signal originated from the reverse transcriptional RNA rather than the plasmid DNA in the transfection mixtures.

### 2.6. Animal Experiments

A total of 150 21-day-old healthy, unvaccinated commercial Three-Yellow chickens (purchased as day-old chicks from a commercial poultry hatchery and raised in isolators) were randomly divided into six groups (n = 25/group) and housed in separated isolators with unlimited access to food and water. Prior to inoculation, all the birds were tested by ELISA to ensure that they were free or negative to the IBDV-specific antibodies as noted in our previous description [58]. The birds of each group were inoculated via the oral route with either sterile phosphate-buffered saline (PBS) or wild-type NN1172, *r*NN1172, *r*NN1172-B87VP1a, *r*NN1172-B87VP1a∆4, or *r*NN1172-VP1∆4, respectively. All the birds were given 0.2 mL/bird of PBS or viruses doses of 1.0 × 10^4^ ELD_50_/bird. Ten birds were randomly selected from each group to be labeled to observe the morbidity and mortality of the groups. Birds were monitored for clinical symptoms for 7 days, and the observations and deaths were recorded daily. Clinical signs were quantified by using the mean symptomatic index (MSI) scores [49] of the birds, calculated daily from 1 to 7 dpi in all the groups of the experiment. To ensure consistency in the evaluation of the MSI, each score was determined independently by two previously trained observers. Five birds per group were randomly selected and euthanatized at 3, 5, and 7 days post-infection (dpi). Bursa samples were removed, and the bursa/body-weight index (BBIX) ratio and IBDV-induced bursal atrophy were determined according to the previous description by Ismail and Saif [59]. Bursae with a BBIX lower than 0.7 were considered atrophied. Bursa samples were collected from dead and sacrificed birds, and one part of them was fixed in 4% neutral buffered formalin solution and used for the histopathological observation according to the routine method in our previous description [60]. The severity of the IBD-induced histopathological lesions was quantified according to Skeeles’ scale for histopathological bursal lesion scores (HBLS) [61]. Another part of the bursa samples was used for RNA extraction, and then the virus quantitative RT-PCR (qRT-PCR) was performed to evaluate the viral replication in vivo according to our group’s previous method [42].

### 2.7. Statistical Analysis

All values were processed using GraphPad Prism 5.0 (GraphPad Software Inc., San Diego, CA, USA) and expressed as means ± SD. Results were analyzed with one-way ANOVA to assess the differences among groups.

### 2.8. Ethical Statement

All procedures in this study were approved by the Animal Welfare and the Animal Experimental Ethical Committee of Guangxi University (No. 2018-gxu-286).

## 3. Results

### 3.1. Construction of the Plasmids of IBDV Genome

The whole genome of the NN1172 strain was amplified, sequenced, and submitted to GenBank (MW245064 and MW245065). With a silent mutation (C501A) of the polyprotein, the genetic tags (unique restriction enzyme site of *Hind* III) were successfully introduced into segment A (Supplementary Materials Figure S1C). The sequencing results revealed that segments A and B of the NN1172 strain were successfully flanked with the sequences of HamRz (58 bp) and HadRz (88 bp). The N-terminal domains of the strains NN1172 and B87 genome were combined to construct a mosaic B fragment. The plasmids pVAX1-mNN1172A, pVAX1-mNN1172B, mNN1172-B87VP1a, mNN1172-B87VP1a∆4, and mNN1172-VP1∆4 were successfully constructed.

### 3.2. Generation and Identification of the Recombinant Chimeric Viruses

All SPF chicken embryos, which were inoculated with the supernatants of cell cultures at 72 h from the primary CEFs transfected with the recombinant plasmids, had died during 3–5 dpi, and severe systemic hemorrhage (Supplementary Materials Figure S1A) was observed as similar to that described previously [62]. The purified PCR products with the embryo homogenate samples digested by the enzyme *Hind* III showed two distinct target fragments (Supplementary Materials Figure S1B), indicating that the viruses harvested were the rescued ones but not the wild-type strain. This genetic tag was not present in the PCR fragment originating from the dsRNA sample of SPF embryos infected with the wild-type NN1172. The full-length genome of segments A and B confirmed that the recombinant viruses, as previously designed, had been successfully rescued, and no unwanted nucleotide change resulting in amino acid substitutions in the genome was found.

### 3.3. The N-Terminal Domain in the RNA Polymerase of NN1172, Substituted by the Corresponding Part of B87, Can Significantly Reduce the Viral Pathogenicity

**Figure 2:**
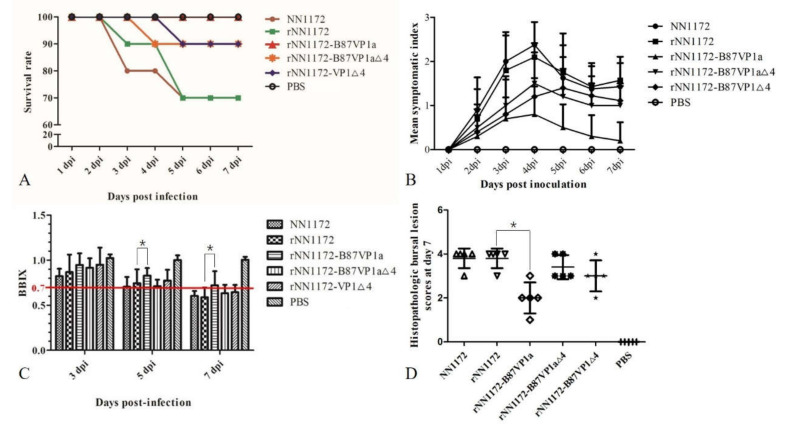
Characteristics of Three-Yellow chickens infected by the rescued virus. (**A**) Percent survival of chickens on different days post-challenge with the recombinant viruses. (**B**) Mean symptomatic index during 1–7 dpi in chickens challenged with mosaic recombinant viruses. (**C**) The bursa/body weight index (BBIX) of chickens challenged with the mosaic recombinant viruses at 3, 5, and 7 dpi. (**D**) Histological bursal lesion scores (HBLS) of chickens challenged with the mosaic recombinant viruses at 7 dpi. * means significantly different (*p* < 0.05).

In the challenge experiments, *r*NN1172 exhibited the same phenotype as its parental strain NN1172. Typical symptoms of acute IBD in the birds (depression, diarrhea, prostration, rough feathers, etc.) were observed during 2–7 dpi, and death occurred during 3–5 dpi (Figure 2A). MSI score showed that *r*NN1172-B87VP1a (maximum 0.8) induced significantly less morbidity than *r*NN1172 and wild-type NN1172 did (maximum 2.1 and 2.38) (Figure 2B). The mortality rate caused by *r*NN1172 was reduced dramatically by substituting NN1172a with the B87a (from 30% in the *r*NN1172 group to 0% in the *r*NN1172-B87VP1a group), and all the bursae from the birds in the *r*NN1172-B87VP1a group were normal with a BBIX maintained above 0.7 (Figure 2C). Except the *r*NN1172-B87VP1a-infected group (HBLS = 2), all the bursae derived from the groups of wild-type NN1172 and *r*NN1172 showed necrosis of lymphocytes, fibroplasias, atrophy of follicles, and follicular depletion with a HBLS maintained above 4 (Figure 2D). Our results indicate that the N-terminus with substitution of the attenuated vaccine strain B87 in the RNA polymerase of vvIBDV can significantly reduce the viral pathogenicity in Three-Yellow chickens.

### 3.4. Substitution of the N-Terminal Domain of the RNA Polymerase of NN1172 by the Corresponding Part of B87 Can Significantly Reduce Viral Replication In Vivo

**Figure 3:**
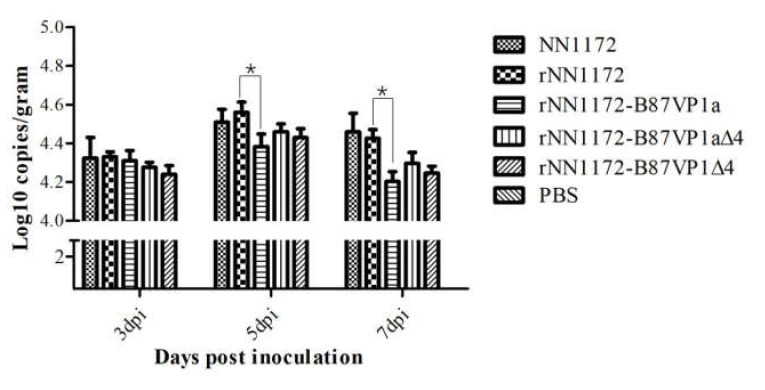
Growth curve of the mosaic recombinant IBDV strains in the infected chickens at 3, 5, and 7 dpi. * means significantly different (*p* < 0.05).

To evaluate viral replication in the experimental birds, the viral genome copy number in each bursa was detected by real-time RT-PCR as described by He et al. [42]. There was no significant difference in the HBLS values between the wild-type NN1172 strain and the rescued parent virus *r*NN1172 at 7 dpi, of which almost all were 4 (Figure 2D). At the same time, higher numbers of viral genome could be detected in the bursa from the birds infected with the rescued virus (Figure 3), thus showing that it replicated well in the bursa. However, compared to the wild-type NN1172 and *r*NN1172 groups, the bursa from the birds infected with *r*NN1172-B87VP1a showed no obvious histological lesions (HBLS = 2). The amount of viral genome detected was the lowest at 5 and 7 dpi (Figure 3), indicating that the viral replication efficiency of the wild-type NN1172 and *r*NN1172 in vivo was reduced significantly by substituting NN1172a with the B87a. The results also revealed that the pathogenicity (damage to bursae) of the IBDV was consistent with the replication efficiency of the virus in vivo (bursae).

### 3.5. Partial Region and V4I Substitutions in the N-Terminal Domain of the RNA Polymerase of NN1172 by the Corresponding Parts of B87 Do Not Completely Lead to Virus Attenuation

As shown in Figure 3, the recombinant chimeric viruses *r*NN1172-B87VP1a∆4 and *r*NN1172-B87VP1∆4 exhibited different phenotypes compared to the wild-type NN1172 strain. MSI scores showed that *r*NN1172-B87VP1a∆4 and *r*NN1172-B87VP1∆4 induced significantly less morbidity than the wild-type NN1172 strain did (Figure 2B). Additionally, the mortalities in groups of birds infected with both the recombinant chimeric viruses were all reduced to 10% and also had a delayed onset of deaths at 4 and 5 dpi, respectively (Figure 2A). The bursae derived from the groups of birds infected with *r*NN1172-B87VP1a∆4 and *r*NN1172-B87VP1∆4 showed atrophy of follicles and follicular depletion with HBLS maintained above 3 (Figure 2D). Our results showed that both the substitutions of partial region and single amino acid residual V4I did not completely lead to virus attenuation even though they both did decrease the pathogenicity.

## 4. Discussion

The emergence of many reassortant/novel variant IBDVs, as an important immunosuppressive disease pathogen, have posed a severe threat to the commercial poultry industry worldwide [39]. Although some efforts have been made to understand the molecular biology of IBDV, the molecular basis for the pathogenicity of IBDV is not fully understood. Previous studies have shown that the segment A of the viral genome, especially VP2 protein, plays an important role in the virulence of IBDV [33,62]. The mutation of VP2 is considered to be one of the factors affecting the virulence evolution of IBDV [35,63]. Recently, many researchers have found that it is not only a single gene that determines the virulence of IBDV but the result of the interaction of multiple genes [29,44,45,64]. The role of VP1 in segment B in viral replication and pathogenicity remains controversial. Nouen et al. found that exchange of different regions of segment B of an attenuated strain Cu-1 with their counterpart 88180 strain could not restore the whole pathogenicity equivalent to the original virus [49], while the N-terminal may play a more important role in virus replication and virulence [50]. Insight into the virulence factors of IBDV will help to develop better measures for the prevention and control of the disease.

Due to the limitations of the reverse genetic system that can efficiently generate recombinant vvIBDV, we restored the virus that had not adapted to cell culture by transfecting plasmids into primary CEF cells and then by passing them into the CAM of the chicken embryo using the previously described method [62]. Using this technology, we successfully generated four viruses, including *r*NN1172, *r*NN1172-B87VP1a, *r*NN1172-B87VP1a∆4, and *r*NN1172-VP1∆4. This method reduces the cumbersome process of producing CEF for virus proliferation in the later period, and a large quantity of viruses can be obtained in a short time. By transfecting DF-1 cells and then injecting the cell culture suspensions into the bursa organs of SPF chickens aged 3 weeks, Yu et al. successfully developed a simple and effective method to rescue vvIBDV using SPF chickens [65]. Compared with the embryo’s CAM approach, this method requires higher skills on the part of the operator. Cubas-Gaona et al. also rescued vvIBDV by transfecting into the DF-1 cells and then transferring the cell culture suspension to the primary chicken bursa cell for virus propagation [66]. However, primary bursa cells are cumbersome to make and not easy to culture. These methods, which all can effectively rescue the vvIBDV, will provide valuable tools for better insight into the molecular virulence factors of non-cell culture adapted IBDV strains.

In order to further clarify the roles of the N-terminal of the polymerase in viral replication and pathogenicity, we conducted a challenge experiment as well as a series of lab-based investigations. Our results with the rescued viruses found that *r*NN1172 showed the same phenotype as its parental strain NN1172, and it demonstrated similar typical symptoms of acute IBD in chickens and the ability to induce morbidity and mortality (30%). They also induced a serious atrophy of the bursa with a BBIX below 0.7 and significant histological lesions with a HBLS maintained above 3, similar to results previously described by other groups [35,62]. The mortality caused by *r*NN1172 in birds was reduced dramatically by substituting the NN1172a with the B87a, from 30% to 0%, and all the bursae derived from the birds in the *r*NN1172-B87VP1a group showed only scattered cell depletion in a few follicles. Interestingly, the recombinant chimeric virus containing the full region of N-terminal domain in the RNA polymerase of the B87 strain was milder than those containing only the VP1a∆4 or VP1∆4 because it significantly reduced the bursal atrophy and histopathological damages. Without changing VP2, partial fragment replacement or V4I substitution of the N-terminal domain does not completely lead to virus attenuation in Three-Yellow chickens, while the substitution of the full region of N-terminal domain of the vvIBDV strain NN1172 can significantly lead to virus attenuation. The N-terminal domain of VP1, as suggested by Gao et al. [50], might play a more important role in the pathogenicity of vvIBDV than the central and C-terminal domains. However, Nouen et al. [49] confirmed that the N-terminal (D1) and especially the central polymerase domain (D2) play an important role in pathogenicity. This indicates that the full regions of the N-terminal of the RNA polymerase may be related to the function or interaction that is more important to the pathogenicity of the recombinant virus, which may offer new targets for their attenuation using reverse genetics. This provides a possibility of the development of new vaccines that retain good immunogenicity (mainly induced by VP2) but reduces pathogenicity to the birds.

Yu et al. found that amino acid V4I substitution influenced viral pathogenicity (the mortality was reduced to 30% in SPF chickens), compared to wild-type vvIBDV. In this study, compared to the parental strain *r*NN1172, the recombinant virus *r*NN1172-VP1∆4, with the substitution V4I in the RNA polymerase, could significantly reduce the pathogenicity by decreasing the mortality from 30% to 10%, which may be related to the reduced ability of viral replication in vivo since our real-time quantification of the viral load in the tissue of bursa was less than that of the parental strain. Three aa residuals (4V, 61I, and 145T) in the N-terminal of the RNA polymerase are conserved in the vvIBDV, while 4I, 61V, and 145N are conserved in the non-vvIBDV [67]. In addition, we found that the substitution of the region of the 5–157 aa residuals in the N-terminal domain of NN1172 with the corresponding part of B87 (this region containing the substitutions of K13T, I61V, L141V, and T145N) can also reduce the viral replication and pathogenicity in birds. It has been confirmed that aa substitutions of I61V and T145N do not affect virus replication and pathogenicity [51]. This suggests that, in addition to the dominant role of V4I substitution in the pathogenicity and viral replication, the K13T and L141V substitutions could also be involved in the pathogenicity and viral replication in birds. The major determinants of function or interaction between K13T and L141V for the viral pathogenicity, however, remain to be understood.

Another interesting finding is that either *r*NN1172 or NN1172 can cause severe atrophy and significant histopathological changes in the bursa, which may be due to the efficient viral replication in the bursa, since a large quantity of viral genomes in each severe damaged bursa could be detected. However, when the NN1172a was substituted by the B87a, the lowest viral genome copy number in the bursa was detected, and no obvious lesion was observed in the histopathological examination of the bursa. These results reveal that the substitution of N-terminus in the RNA polymerase of vvIBDV with the counterpart of the attenuated IBDV can significantly reduce the replication efficiency of the virus in chickens, and the pathogenicity (damage to bursae) of the virus was consistent with the viral replication efficiency in vivo (bursae). Gao et al. also found that the N-terminal domain of VP1 from vvIBDVs had a negative effect on viral replication in vitro while exhibiting a positive effect on viral replication in vivo [50]. However, Nouen et al. [49] found that the association of the central polymerase domain (Dc) and non-coding regions (NCRs) at the 5′ and 3′ ends of the 88,180 strain increased the titer of virus at 4 dpi, but it did not result in an increased pathogenicity in chickens. This difference may be caused by the different background of segment B used in the different experiments, and Yu et al. [51] suggested the mechanisms by which strains with different backgrounds involved in viral virulence enhancement and attenuation might be different. Our result can also explain some of the experiments that reported the tissue culture adapted virus can replicate well in CEF cells and cause obvious cytopathic effects, which may be due to the high-efficiency replication of the virus on CEF cells [62]. In addition, a delayed replication capacity and onset of death in birds have also been described in our study, which could be due to the weakening of some interactions described to occur between both genome segments and/or the proteins they encode [49,68]. The reduction in pathogenicity in vvIBDV strains needs to be further studied, and it should be emphasized that part of the viral pathogenicity can also be determined by the interactions of VP1 and other viral proteins with host immune responses, either by delaying protective immune responses or by inducing unregulated and excessive immune responses, which favor immunopathological changes in the host organism.

In conclusion, our results have demonstrated that the full-region substitution in the N-terminal domain of the RNA polymerase of vvIBDV with the counterpart of the attenuated strain can lead to significant attenuation in the viral replication and pathogenicity, while the substitutions of partial region or single amino acid V4I in the N-terminal domain of the RNA polymerase does not completely lead to virus attenuation, although it significantly reduces its pathogenicity. Our results revealed that the pathogenicity of the IBDV was consistent with the viral replication efficiency in vivo (bursae).

## Figures and Tables

**Table 1:** The primers used for homologous recombinations and mutations in the genome and construction of mosaic B segments.

Primers	Primer Sequences (5′–3′) ^a^
A3U/B3U	tagtccagtgtggtg***gaattc***TGTTAAGCGTCTGATGAGTCCG
A3L/B3L	tgctggatatctgca***gaattc***CGCCCTCCCTTAGCCATC
ADTB-F	ACTGTCCT**a**AGCTTGCCCACATCATATGATCT
ADTB-R	GGCAAGCT**t**AGGACAGTTACCCCTTCCCCTAC
ZTB87NF	ACCTACATGGGGCAAGCGA
ZTB87NR	GGTGGCAGAATCATCAAGAAGAG
B87NF	ttcttgatgattctgccaccATGAGTGACATTTTCAACAGTCCAC
B87NR	gtcgcttgccccatgtaggtCCCACTTCCATAGGCCTTGTC
ZTB87NBFR	GTTGAATACGTCACTCATGGTGGC
B87NBFF	ccatgagtgacgtattcaacAGTCCACAGGCGCGAAGC
WD4TBF	CCACCATGAGTGAC**a**TATTCAACAGTCCACAGGCGC
WD4TBR	TAtGTCACTCATGGTGGCAGAATCATCAAGAA

^a^ A repeated sequence of lowercase letters at the 5‘ end constitutes a homologous arm. The lowercase bold letters indicate the mutation site, and the italic bold letters indicate the restriction enzyme site.

**Table 2:** Segments co-transfected with various combinations and the rescued viruses.

Segment A	Segments B	The Rescued Viruses
pVAX1-mNN1172A	pVAX1-mNN1172B	rNN1172
pVAX1-mNN1172A	pVAX1-mNN1172B-B87∆VP1a	rNN1172-B87VP1a
pVAX1-mNN1172A	pVAX1-mNN1172B-B87VP1a∆4	rNN1172-B87VP1a∆4
pVAX1-mNN1172A	pVAX1-mNN1172B-B87VP1∆4	rNN1172-B87VP1∆4

## Data Availability

Data is contained within the article or supplementary material. The data presented in this study are available in insert article or supplementary material here.

## Supplementary Materials

The following are available online at https://www.mdpi.com/1999-4915/13/1/107/s1, Figure S1: Generation and identification of mosaic viruses, Table S1: Primers used for the amplifications of the full-length genome of IBDV strains, Table S2: Primers were synthesized to introduce the ribozyme structure and unique restriction enzyme (*Hind* III) site in the genome of the recombinant IBDV strains.
