# Supplementary material for: The Full Region of N-Terminal in Polymerase of IBDV Plays an Important Role in Viral Replication and Pathogenicity: Either Partial Region or Single Amino Acid V4I Substitution Does Not Completely Lead to the Virus Attenuation to Three-Yellow Chickens
Source: Viruses. 2021 Jan 14;13(1):107. doi: 10.3390/v13010107 (PMC7828667; doi:10.3390/v13010107)
Supplement: Supplementary file 1 [file viruses-13-00107-s001.pdf]

Table S1. Primers used for the amplifications of the full-length genome of IBDV strains

Primers[a]	Primer sequences (5′- 3′)
A1-F	GGATACGATCGGTCTGACCCCGGGGGAGTC
A1-R-fusion	CTTCAGGGGAGAGTTGAGGTC
A2-F-fusion	GACCTCAACTCTCCCCTGAAG
A2-R	GGGGACCCGCGAACGGATCCAATTTGGGAT
B1-F	GGATACGATGGGTCTGACCCTCTGGGA
B1-R-fusion	TCTAGGTCAATTGAGTACCAC
B2-F-fusion	GTGGTACTCAATTGACCTAGA
B2-R	GGGGGCCCCCGCAGGCGAAGGCCGGGGAT

Table S2. Primers were synthesized to introduce the ribozyme structure and unique restriction enzyme (*Hind* III) site in the genome of the recombinant IBDV strains

Primers	Primer sequences (5′-3′)[a]
AF01-P5	TGAGGACGAAACTATAGGAAAGGAATTCCTATAGTCGGATACGATCGGTCTGAC
AR01-P6	CGGACCGCGAGGAGGTGGAGATGCCATGCCGACCCGGGGACCCGCGAACGGATC
AF02-P7	CCGGAATTCTGTTAAGCGTCTGATGAGTCCGTGAGGACGAAACTATAGGAAAG
AR02-P8	GAGTGGACGTGCGTCCTCCTTCGGATGCCCAGGTCGGACCGCGAGGAGGTGGAG
AR03-P9	CGGGGTACCCGCCCTCCCTTAGCCATCCGAGTGGACGTGCGTCCTCCTTC
BF01-P10	TGAGGACGAAACTATAGGAAAGGAATTCCTATAGTCGGATACGATGGGTCTGAC
BR01-P11	CGGACCGCGAGGAGGTGGAGATGCCATGCCGACCCGGGGGCCCCCGCAGGCGA
BF02-P12	CCGCTCGAGTGTTAAGCGTCTGATGAGTCCGTGAGGACGAAACTATAGGAAAG
BR02-P8	AR02-P8
BR03-P13	CGGGGTACCCGCCCTCCCTTAGCCATCCGAGTGGACGTGCGTCCTCCTTC

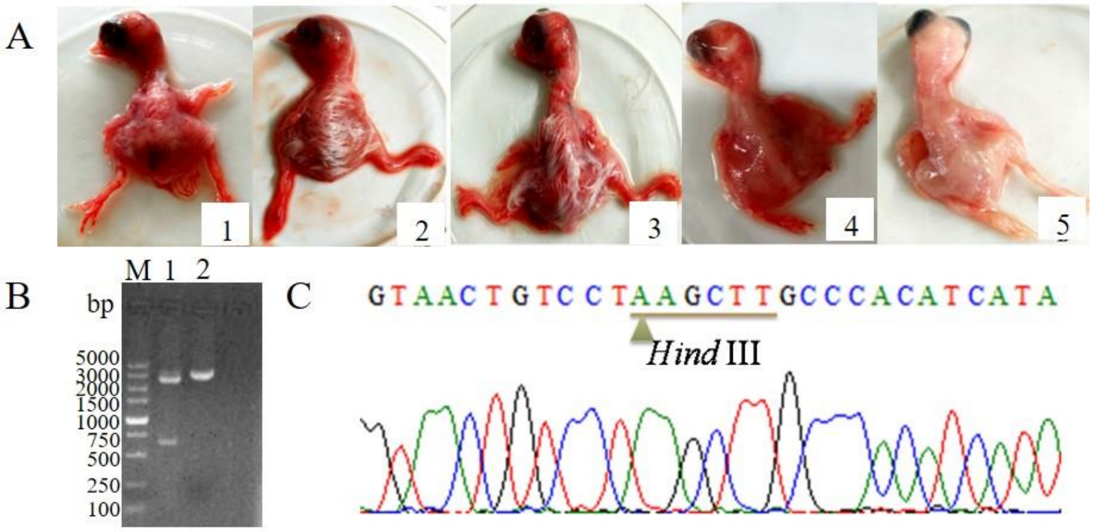

Figure S1. Generation and identification of mosaic viruses. **A**, the pathogenic changes of the SPF chicken embryos inoculated with the recombinant viruses: 1 *r*NN1172-B87VP1a; 2 *r*NN1172-B87VP1aΔ4; 3 *r*NN1172-VP1Δ4; 4 *r*NN1172; 5 The empty plasmid. **B**, The purified PCR products digested by the enzyme (*Hind* Ⅲ) showed two distinct target segments (2 629bp and 631bp) of the rescued viruses: M markers; 1 The purified PCR products digested by the enzyme (*Hind* Ⅲ) of the rescued viruses; 2 The purified PCR products digested by the enzyme (*Hind* Ⅲ) of the wild-type viruses; **C**, The genetic tags (unique restriction enzyme site *Hind* III) were successfully introduced into the segment A used to identify the recombinant viruses.
